# After a Fracture of the Limb the Nails of the Hand on the Same Side Cease Growing

**Published:** 1898-11

**Authors:** 


DR. REID again draws attention to the fact that for some days after a fracture of the limb the nails of the hand on the same side cease growing. As soon as the bones begin to knit firmly the nails begin to grow.—*Mass. Med. Jour.*, 1993, *No.* 13, *page* 488.

